# Stories of paediatric palliative care: a qualitative study exploring health care professionals’ understanding of the concept

**DOI:** 10.1186/s12904-022-01077-1

**Published:** 2022-10-22

**Authors:** Kirsti Riiser, Heidi Holmen, Anette Winger, Simen A. Steindal, Charlotte Castor, Lisbeth Gravdal Kvarme, Anja Lee, Vibeke Bruun Lorentsen, Nina Misvaer, Elena Albertini Früh

**Affiliations:** 1 Department of Rehabilitation Science and Health Technology, Faculty of Health Sciences, OsloMet - Oslo Metropolitan University, St. Olavs plass, NO-0130 Oslo, PO Box 4, Norway; 2 Department of Nursing and Health Promotion, Faculty of Health Sciences, OsloMet - Oslo Metropolitan University, St. Olavs plass, NO-0130 Oslo, PO Box 4, Norway; 3 Lovisenberg Diaconal University College, Lovisenberggt. 15b, NO-0456 Oslo, Norway; 4 Department of Health Sciences, Faculty of Medicine, Lund University, SE-221 00 Lund, Box 157, Sweden; 5 Division of Paediatric and Adolescent Medicine, Oslo University Hospital, NO-0424 Ullevål, Nydalen, Oslo, PO Box 4950, Norway; 6 Department of Nursing, Faculty of Health Studies, VID Specialized University, NO-0319 Vinderen, Oslo, PO Box 184, Norway

**Keywords:** Paediatric palliative care, Palliative care, Health care professionals, Life-threatening conditions, Life-limiting conditions, Concept, Storytelling, Thematic analysis

## Abstract

**Background:**

By sharing patient stories, health care professionals (HCPs) may communicate their attitudes, values and beliefs about caring and treatment. Previous qualitative research has shown that HCPs usually associate paediatric palliative care (PPC) with death or dying and that they find the concept challenging to understand and difficult to implement. Attending to HCPs’ stories may provide a richer account of their understanding of PPC. Thus, the aim of this study was to explore PPC stories narrated by HCPs to gain increased insight into their understanding of what PPC entails.

**Methods:**

This qualitative study collected data from four focus group interviews with 21 HCPs from different units in two Norwegian hospitals. Stories told by the HCPs to illustrate their comprehension of PPC were analysed following thematic analysis procedures.

**Results:**

Four themes were identified illustrating what PPC meant to the participants: creating spaces for normality, providing tailored support for the family, careful preparations for saying goodbye and experiencing dilemmas and distress. The stories centred on family care, particularly relating to dramatic or affective situations when the death of a child was imminent.

**Conclusion:**

The stories reflect how the HCPs view PPC as a specific field of health care that requires particular professional sensitivity, including good communication, collaboration and planning. Thus, the HCPs in this study demonstrated knowledge about the core qualities needed to succeed in PPC. However, similar to previous research, the stories illustrate that how HCPs speak about PPC is strongly associated with end-of-life care, and by that the HCPs do not capture the breadth of the PPC concept. The findings highlight the importance of increasing knowledge about the meaning and content of PPC among HCPs in order to maintain quality of life for all children with life-limiting or life-threatening conditions throughout their illness trajectory.

## Background

Storytelling is a fundamental part of being human. Through stories information can be shared in a way that creates an emotional connection and by that provides a deeper understanding of the storyteller’s experiences. Thus, storytelling has the potential to bring new perspectives to the Table [1]. When telling stories about specific events from patients´ lives, health care professionals (HCPs) not only describe the patients’ medical situations. By sharing patient stories, HCPs also communicate their attitudes, values and beliefs about caring and treatment [2]. The present study used storytelling as a methodological approach to investigate HCPs’ understanding of paediatric palliative care (PPC). According to the World Health Organization (WHO), PPC should be the standard of care for children with life-threatening or life-limiting conditions and should be integrated with prevention, early diagnosis and treatment [3, 4]. PPC is defined as “the active total care of the child’s body, mind and spirit, and [this] also involves giving support to the family” [5]. Children (0–18 years) and their families should be provided with interdisciplinary care aiming to promote their physical, psychological, social and spiritual well-being [4]. Early implementation of PPC through active and integrated support is endorsed as beneficial to the well-being of the child and the family [3, 6, 7]. HCPs may display good knowledge of the principles of palliative care, but their comprehension of the concept, their way of thinking and their reported reasons for referrals to PPC demonstrate that they associate PPC with death and dying [8, 9]. Research reports that HCPs find the concept challenging to understand and implement and that it evokes negative emotions [10–12]. Consequently, early integration of PPC may be delayed and many children are not referred to PPC until late in their illness trajectory [12]. Among the barriers reported for implementing PPC at an early stage are uncertainty and confusion about the concept, as well as stigma related to the term [10, 13]. In our recent study of interdisciplinary HCPs’ understanding and implementation of PPC, we found that the participants viewed the concept as unfamiliar and not meaningful [11]. HCPs associated PPC with death and dying and used words like scary, burdensome, dramatic and lack of active treatment to describe their comprehension of the concept [11].

Attending to stories of HCPs´ clinical experience with children and their families may provide further insights than solely through answering questions and thus be a valuable approach to gain an even richer account of their understanding of PPC. Thus, the aim of this study was to explore stories of PPC narrated by HCPs to enhance our knowledge of their understanding of what PPC entails.

## Methods

### Design

This study included qualitative data obtained through focus group interviews with HCPs and emerged from a project exploring HCPs’ understanding of PPC [11]. The project was not originally designed to elicit narratives; however, during the focus groups, the HCPs told stories or gave specific examples to illustrate their comprehension of PPC. We decided to subtract these stories and all the utterances made from the initial material to provide examples or illustrate points when reflecting on what PPC is. Thus, the data was not a part of the material analysed in the first article. We explored the stories in more depth, with the assumption that this may provide an enhanced understanding of what HCPs comprehend when they talk about PPC. The reporting of the study was guided by the consolidated criteria for reporting qualitative research [14].

### Participants

HCPs from three paediatric units in two hospitals were recruited using purposeful sampling, including a variety of professions. Both hospitals are located in Eastern Norway. The units include different departments for children aged 0–18 with a wide range of diagnoses. A total of 21 participants, of whom 19 (90%) were female, participated in 4 focus groups including 2–9 participants (Table 1). Most of the participants had worked with children with life-threatening and life-limiting conditions for more than 10 years. Only two had fewer than two years of experience and some had more than 20 years. Without giving any reason, four HCPs from one of the units withdrew their consent, which is why one of the groups ended up including only two participants. The other three groups included four, six and nine participants. We have no information about the HCPs who declined invitations to participate.

**Table 1:** Participants

Focus group number	Number of participants	Professions
1	4	Paediatric nurses (2) Nurses (2)
2	9	Clinical social worker Oncology nurse Paediatric nurses (2) Nurse Chief Physician Specialist in psychology Paediatrician Physiotherapist
3	2	Physiotherapist Paediatric nurse
4	6	Nurses (3) Clinical social worker Chief physician Medical doctor

### Data collection

The four focus group meetings were convened between November 2019 and February 2020 in a suitable location at the hospital where the participants worked. The focus groups were moderated by pairs of researchers (AL and KR or LGK and EAF): one researcher led the conversations, and the other assisted and took notes during the focus group. All the moderators are female HCPs with a PhD or a master’s degree. None had any prior professional or private relationship with the participants. All the focus groups started with a short presentation of the aim of the study and the researchers’ background and research interests. A semi-structured interview guide was used to focus the conversation and keep it within relevant topics. The guide was developed based on previous research and included open-ended questions related to the concepts of PPC, alleviation and end-of-life care. The participants were also encouraged to illustrate these concepts by sharing their experiences from working with the children and their parents [10]. The interview guide was not piloted, but the questions were discussed with a reference group including HCPs, service user representatives and researchers to facilitate relevant and clear questions. The focus groups lasted from 45 to 90 min and were audio-recorded.

### Data analysis

The focus group interviews were transcribed verbatim by a professional transcriber and imported into the qualitative software package NVivo 12 to aid the analysis. The data analysis was undertaken following a thematic analysis procedure [15]. We applied an inductive approach, meaning that the data were coded without trying to fit a pre-existing coding frame [15]. First, the entire data set was screened for stories of PPC. In addition to the stories shared on request, we included all utterances made to provide an example or illustrate a point when reflecting on what PPC is. The transcripts were read by all the authors to obtain an overall first impression of their content. Next, three authors (KR, HH and AW) jointly coded the transcripts section by section aiming to capture the essence of the data before grouping the codes into semantically related themes determined by perceived patterns. A preliminary set of themes with codes was presented to the other authors, who all provided their comments. Based on the feedback, the first and last authors (KR and EAF) reviewed and refined the themes and validated them against the data set. To tell the story of each theme, a detailed analysis of each theme was written. Lastly, the themes were named with the aim of providing an understanding of each theme’s content [15]. Quotations from the focus groups used in the article were translated into English by the researchers in collaboration. Minor alterations were made to the text to ensure anonymity.

Dependability was enhanced by being explicit about the data analysis process and how this was systematically carried out. Credibility was enhanced through investigator triangulation in the data analysis [16]. All the researchers were involved in one or more steps of the analysis, and we challenged each other to provide alternative interpretations and reach intersubjectivity. All the authors are skilled researchers, and most have extensive experience in qualitative research.

### Ethical considerations

The project was reviewed by the Norwegian Data Protection Official for research, who concluded that it was in accordance with the Personal Data Act (reference number 935,944). The local data protection officers at the two participating hospitals approved the study. The study participants were HCPs, and the project did not collect data about health and illness. Thus, the project did not require permission from a regional committee for research ethics. The participating HCPs received information about the aim of the study and the procedure for data collection and gave written consent to participate after being reassured about confidentiality issues and their right to withdraw.

## Results

Following the analysis, four themes were identified illustrating what PPC entails to the participants: *creating spaces for normality*, *providing tailored support for the family*, *careful preparations for saying goodbye* and *experiencing dilemmas and distress* (Table 2).

**Table 2:** Themes and corresponding codes and examples of quotes from the stories

Themes	Codes	Quotes
Creating spaces for normality	Everyday life and play Supporting normality	*Like that one time, we hid under the blanket because she did not want to talk about her disease. When the doctor tried, the girl hid under the blanket, and I asked to join her. So we stayed there together. We kind of had an alliance. It is about seeing them exactly where they are.*
Providing tailored support for the family	Providing knowledge and support Providing extraordinary care Layer upon layer Creating safety	*The parents felt safe when coming here, and they felt safe to leave the room. They could breathe a little while they knew she was taken care of.*
Careful preparations for saying goodbye	Making arrangements for saying goodbye Being together Capturing the right moments for difficult conversations Realizing that death is imminent	*And we sat there at the outpatient clinic and I thought to myself that I have to tell them…because I do not know how long he would live, so we should talk about that. And at that moment I was given an opportunity to say that if he came back with an infection we might not be able to do…to save him…or he could end up on a ventilator and that is not necessarily a good idea because we cannot get them off. So we talked about that.*
Experiencing dilemmas and distress	Conflicting wishes Dilemmas relating to treatment	*It was obvious to everyone else that the child’s condition was very severe and that she would never recover, and then the parent says “well, that is just what* *you* *believe!” (…) If the parents want treatment, when we have no treatment to offer, there is always someone that makes promises without scientific foundation.*

### Creating spaces for normality

The HCPs seemed attentive to having the child with a life-threatening or life-limiting illness at the centre of their care. Several participants spoke about the importance of supporting a sense of normality and mentioned that the parents as well as the child aimed for ordinary days at home, school or kindergarten: “I believe that most parents want their child’s days to be normal and good until the end and that it’s not going to be something else”. (Group 3)

Stories were told that illustrate how the HCPs managed to create spaces for play and fun for the child despite working in a hospital and being responsible for clinical procedures. They gave examples of how they used medical equipment as props for play and how these playful situations opened up conversations with the child about sensitive and important topics. In one story, the nurse explained that they made the hospital stay so engaging that the child looked forward to treatment and felt sorry for leaving. Another nurse elaborated on a playful moment with a little girl with a life-limiting illness engaging in imaginary play:

*…and then the girl says: “At my prom, I want a dress like that”, and I said “Really? Wow, that’s cool”, and then she kind of realizes that she’s never going to a prom and then she says: “Well, well…it would have been cool, right?” and I responded “Yeah, it would have been really cool” (Group 2).*

The nurse reflected on the importance of being present in the moment and allowing the child to enjoy the thought of adolescence even though her life expectancy was very limited.

### Providing tailored support for the family

The participants spoke about different ways of providing support through adjusted information and tailored care. In their stories, they emphasized the importance of sufficient, timely information. One participant described how a mother once said that she did not understand that her child could die while being on a ventilator and that the lungs could still collapse. The participant had carried this mother’s utterance with her ever since, and she pointed out that making sure that parents understand why their child actually dies is significant. Another participant described how important it is to be able to recognize when the parents communicate a need for more knowledge and to provide accurate information when it is called for. However, the participants also referred to challenging situations in which the parents and HCPs were not in step with each other. One participant admitted that if HCPs believe the parents are informed about the child’s situation and suddenly realize that they are not, this may cause delays in procedures and irritation:

*I try to be observant to this, both in myself and others. It’s important to avoid a moralizing attitude: ‘Do they [the parents] still not understand?’ Because it creates hassle and annoyance when the parents are not sufficiently informed and up to date on the child’s situation. (Group 2)*

The participants gave several examples of how they adjust and tailor their care for families to make them feel safe in acute as well as more stable situations. They spoke about the importance of working as a team around the family. One participant described it as creating layers upon layers of love around the child. This expression derived from a particular story about a child who was going to be placed in foster care but was too sick to leave the hospital. With help from the HCP, the child’s birth mother and foster mother moved into the children’s ward. With support from the multidisciplinary team, they cared for the child together during the last weeks of the child’s life.

*…and the foster mother stepped aside to let the birth mother hold the child close, day and night (…) and the baby received so many layers of love (…) And the HCPs were amazingly professional and put things aside to let it happen. (Group 2)*

Later, the birth mother explained that, despite the great tragedy, these had been the finest weeks of her life. This situation was resource-demanding for the unit, but all the HCPs stood out as highly professional and were willing to make an extra effort in the extraordinary situation.

Going that extra mile for children and their families was a recurring theme in the HCPs’ stories. Witnessing severe and distressing situations seemed to make the HCPs mobilize to make the days, weeks and months as good as possible for the child, their parents and their siblings. Many of the participants’ stories were about creating safe spaces for families at the hospital, giving the child, parents and siblings moments to breathe. There were also examples of systems failing to support the families adequately and lack of coordination resulting in disorganized care; however, most stories were examples of how HCPs managed to work together, emphasizing the importance of shared information. One participant said:

*One of the finest moments that I’ve witnessed was when a nurse who knew the family well took the initiative to play with an older sibling of a child who would soon die. The nurse took the sibling outside in the hallway, and they talked and played it out. They had a good relationship. The nurse knew very well what was about to happen because she had been in the room when the message about the child’s impending death was given. The more people working around the family, the more important it is that we have the same information and work as a team. (Group 4)*

### Careful preparations for saying goodbye

Most of the stories emerged from critical situations or when the child’s illness was worsening, especially about initiating advance care planning with the end of the child’s life in mind. Some described the importance of seizing that particular moment when the parents and the child are ready and receptive to these difficult conversations. The HCPs gave examples of when the parents themselves took the initiative, but in most of the stories, the HCPs took the lead. However, as underlined by one participant, engaging in such dialogue requires that the HCPs are present and that they tread carefully: “If you’ve established a good relationship, moments will occur if you’re observant and willing to enter these conversations. For me, this is part of palliative care”. (Group 2)

With the children, such conversations could take place during play, and difficult topics could be touched upon without needing to make eye contact. It became evident through the HCPs’ stories that these conversations were tough and that it was sometimes difficult to control their own feelings.

*These conversations are really hard, it is as if you are about to start crying yourself. But I think it is really, really important [to talk about it], because we have reached the moment when it might happen. (Group 3)*

Through their stories, the HCPs emphasized the need for advance care plans when the risk of severe incidents increased. The advantages of such plans are to secure continuity and ensure that all important information is available for all the HCPs involved in a possible emergency situation. However, these plans should also be made for the family members so that they know how to act if critical events occur. In several stories, the HCPs described how the care plans included strategies and measures for staying at home as much as possible. One example was given of how specialist care and primary care cooperated, where the child was provided with advanced home care, and the child and the family shifted between being at the hospital and being at home. The family in this story expressed a strong wish to let the child die at home, and the HCPs made great efforts to accommodate this wish. The participant described how all the necessary equipment was installed in the family’s house:

*It was arranged for him to stay at home. He received pain relief, and I believe the community nurses cared for him, and he spent his last 2–3 weeks in the living room together with his siblings and parents and they all slept together in the same bed. They were kind of… they were together.” (Group 1)*

Making practical arrangements to facilitate a proper farewell in a safe environment together was a prioritized task. The parents and siblings could focus on being there for the child and for each other while the medical team provided care and pain relief for the child.

### Experiencing dilemmas and distress

While many stories and examples evolved from cases where the HCPs explained that they managed to provide support and good care, there were also stories about conflicts with parents and distressful experiences. Some HCPs referred to conversations about how to handle a dramatic worsening of the child’s condition (e.g. decisions about not putting the child on a ventilator or stopping medications) and how this had provoked anger and frustration among parents. One HCP described one such situation:

... *and then there were discussions about withdrawing antibiotics, and we had to tell the parents, and one of them was really devastated and said, ‘You*’*re giving up on my child!’ whereupon the doctor kindly responded, ‘No, we’re not, we are comforting her during her last days’”. (Group 2)*

In some cases, the participants noted that it was the HCPs and not the parents who pushed too hard by suggesting a new treatment, which turned out to result in complications or negative side effects. Another story illustrated the tension that can occur between HCPs and parents when the child is expected to die within a short time. In this case, one HCP accused a parent of hastening the baby’s death by withholding nutrition. This resulted in a deep conflict among the staff, where some supported the nurse and some sympathized with the parents. Eventually, external counselling was required to resolve the dispute among the staff. After the baby died, the parents reported that the whole situation had added even more to their grief. The participant described this as an ethically demanding situation, and, in retrospect, she was struck by how good intentions can lead to conflict and how emotionally involved one may become.

Examples were also given of parents who refused to acknowledge that their child had a life-limiting disease and instead of planning for the end spent all their time searching for a cure. The HCPs experienced it as particularly hard to witness how false hope was being sold through experimental treatment and that parents were willing to squander that precious, limited time left of their child’s life:

*There was a family that I was never in a position to speak with, because their attention was always directed at something else. It was impossible to talk to them about anything, because they were always focusing on going off somewhere to find a cure. (Group 2)*

In these stories, the parents searched for opportunities to travel far with their child to try experimental treatment, resulting in long periods away from home and separation between family members. The participant found it challenging that the parents were never available to make plans for good palliative care.

## Discussion

As a means of enhancing our knowledge of HCPs’ understanding of what PPC entails, the present study aimed to explore HCPs’ stories about PPC. Our findings are in line with the findings of previous studies that HCPs associate PPC with advanced illness or more acute phases towards the end of the child’s illness trajectory [8, 10, 11]. The HCPs’ stories circled around family care, particularly relating to dramatic or affective situations when the death of a child was imminent. The stories reflected how the HCPs view PPC as a specific field of health care that requires particular professional sensitivity, including good communication, collaboration and planning. Thus, the HCPs in this study demonstrated knowledge about the core qualities needed to succeed in PPC [17].

The potentially complicated illness trajectories in children with life-limiting and life-threatening conditions make early integration of palliative care particularly beneficial [3, 4]. The stories in this study rarely touched upon topics related to earlier palliative phases like living everyday life with a life-limiting disease or coordinating follow-up at home or school. Possible consequences for late referral to palliative care are that symptoms may be unaddressed for longer and psychological needs may be neglected [18]. Bearing in mind that the participants in the present study all worked in specialist healthcare, it is reasonable that most stories revolved around acute and life-threatening situations at a hospital. However, according to the WHO’s guidelines and the guidelines for PPC in Norway, specialist health care and primary health care services should be involved in the entire process from diagnosis onwards, assuring overall and coordinated follow-up of the child and the family [3, 4]. The national guidelines emphasize the importance of continuous mapping, evaluation and adjustment to the family’s needs and that this is a shared responsibility between primary and specialist health care [3]. Over the last years interdisciplinary PPC-teams have been established in children’s wards throughout the country, aiming at provision of care regardless of whether the child is at home, in institutions or in hospitals. In some of the larger cities in Norway, the ‘Hospital at home’ provides specialized health care in the child’s home. Many families express a strong wish for home care [19]. Only a small number of the stories included examples of cooperation with community care, enabling the families to stay at home. This indicates that the HCPs either had few such experiences of collaboration outside the hospital or that the collaboration they engaged in did not fall within their conception of PPC and that they did not regard such work as palliative care. This demonstrates a need for clarification of the distinction between basic and specialized PPC. Basic PPC is to some degree integrated into standard care of children with life-limiting and life-threatening conditions in Norway, but specialized PPC is a relatively new field of medical expertise that was brought to public attention with the first national guidelines in 2016 [3].

While few stories in this study exemplified how HCPs work cooperatively outside the hospital, several stories described the necessity and value of working as a team *within* the hospital. PPC is defined by the WHO as total care of the child’s body, mind and spirit, which also includes care of the family [4]. Thus, interdisciplinary teamwork is essential for the provision of PPC. To evaluate and alleviate the distress of the child and the family and promote their well-being throughout the illness trajectory, multiprofessional teams need to work in an interdisciplinary way. Some participants emphasized the importance of equal access to information and shared understanding of a child’s and family’s situation within the health care team. Although each team member has a particular scope of practice and responsibility, a mutual understanding based on shared experiences and information enables mutual decision-making [20], which can be particularly important in acute or stressful situations. An interdisciplinary team approach, which includes the child and/or the parents, is essential for achieving holistic and comprehensive care encompassing the complex needs of the patient and the family [21]. One study suggests that HCPs in palliative care teams enhance their ability to see the patient from a holistic perspective by sharing impressions and discussing the patient and family’s situation within the team [22].

Even though the HPCs’ stories were not about early PPC stages, they do illustrate important elements of *later* phases, as described in national and international guidelines [3, 4]. Many of the stories accompanied the categories identified in our previous paper relating to family-centred care and preparing for the death of a child [11]. The stories show that the participants recognize the importance of having a family-centred care perspective. The HCPs’ attention is very much drawn to how they can best support the family. In palliative care, family-centred care is defined as seamless continuity addressing patient, family and community needs related to terminal conditions through interdisciplinary collaboration [23]. Family-centred care has been described as containing the principles of information sharing, respecting and honouring differences, partnership and collaboration, negotiation and care in the context of family and community [24]. A child’s and family’s needs and preferences vary. Thus, it is not possible to deliver family-centred PPC with a one-size-fits-all approach. Among the stories told were examples of how HCPs stood out as creative, flexible and responsive to the child’s or the parents’ specific needs through adjusting their information and care for the child and the family. Meanwhile, the participants also emphasized the need for structured advance care planning, particularly when death is approaching. Engaging in advance care planning requires that future scenarios be discussed with families [25]. Coming to a realization that death is inevitable and close is a process for those involved, and families as well as HCPs reach this understanding at different times and paces [26, 27]. Several stories evinced that having these conversations is demanding for HCPs, that timing is key and that insufficient communication may have severe consequences for the parties involved. Conversations about future care and end-of-life planning were mostly initiated by HCPs and less often by the parents in the stories we collected, which corresponds with previous research findings [26]. Several frameworks and tools have been developed to address advance care and end-of-life planning, some of which are specifically developed for PPC [28]. However, no such tools were mentioned by the participants. Instead, their stories indicated that they relied on their experience and professional tact when engaging in dialogues about palliative care. This may reflect the status and development of PPC in Norway at the time of this study. Advance care planning as a specific method has just recently been integrated into the guidelines of the Norwegian Association of Paediatricians [29].

The overall aim of PPC is to ensure a child’s quality of life throughout the illness trajectory [4]. Cherishing moments of joy and aiming for days of normality for the children were spoken of as important by the participants. The support of HCPs in achieving this, has been shown to be of great importance to parents as well [30]. For children, normality equals playing. The participants’ stories of how they provided children at the hospital with time and opportunities to play illustrate that the participants recognize its value. Playing moves the child out of time, allowing them to be absorbed in the moment and making room for absolute presence while at the same time giving the child an increased understanding of their existence [31]. This was beautifully illustrated in the story about the girl who knew she would not live to experience her prom but still allowed herself to joyfully engage in play about it. As death approaches, time increases in value. Witnessing how children capture time in play may help parents as well as HCPs broaden their understanding of what PPC comprises.

## Strengths and limitations

Using stories as a basis for analysis is a strength of the present study. When we tell stories we use language available and familiar to us and to our audience [1]. While HCPs’ understanding and experience of PPC has been investigated through qualitative interview studies, few, if any, have used storytelling as a research tool to explore their insights. Compared to other qualitative research methods, stories can generate more nuanced, contextualized and culturally reflective information [32]. However, participating in focus groups instead of individual interviews may have limited the HCPs’ time and opportunity to elaborate on their stories. In only one of the four focus group interviews did the interviewer explicitly ask the participants to tell a story or give an example from practice. This is an important limitation. If the same request had been given to the remaining groups, we might have collected richer data. Nevertheless, the participants in all the focus groups spontaneously used their experiences to elaborate on their comprehension of PPC through stories and examples, with little prompting required from the interviewers. Some chose to supplement their colleagues’ stories or follow up with their own version of the same case, while at other times listening to a story served as a cue for a new story mirroring or contrasting with the former. The stories stood out as significant to the storyteller, and it was noticeable that they had been told and reflected over before. This underlines the significant potential of stories to capture the participants’ lived experiences. One limitation to be noted is that the sample was predominantly female and thus the results may be less representative for male HCPs.

## Conclusion

In this study, we aimed to explore stories of PPC as narrated by HCPs to enhance our knowledge of their understanding of what PPC is. In our previous paper, we discussed that although the HCPs were hesitant to use the term PPC, they found themselves responsible for delivering PPC [11]. The HCPs’ stories supported this by highlighting key elements of PPC, such as interdisciplinary teamwork family-centred care and advance care planning. However, similar to findings in our first paper [11] and previous research [8–10], the stories illustrated that HCPs’ reflections about PPC did not capture the entire concept of PPC, as they were strongly associated with end-of-life care. This underscores the importance of increasing knowledge about PPC among HCPs in Norway. A consolidated recognition of the meaning and content of PPC is necessary to maintain quality of life for all children living with life-limiting or life-threatening conditions.

## Data Availability

The datasets generated and analysed during the current study are not publicly available due to legal restrictions. Data are however available from the authors upon reasonable request and with permission of the Norwegian Centre for Research Data (NSD) and the hospitals’ Data Protection Officers.

## Abbreviations

**PPC**: Paediatric palliative care
**HCP**: Health care professional
